# Water mediated growth of oriented single crystalline SrCO_3_ nanorod arrays on strontium compounds

**DOI:** 10.1038/s41598-021-82651-0

**Published:** 2021-02-09

**Authors:** Junsung Hong, Su Jeong Heo, Prabhakar Singh

**Affiliations:** 1 Department of Materials Science and Engineering, University of Connecticut, Storrs, CT 06269 USA; 2 Present Address: Department of Materials Science and Engineering, Northwestern University, Evanston, IL 60208 USA; 3 Present Address: Materials Science Center, National Renewable Energy Laboratory, Golden, CO 80401 USA

**Keywords:** Mineralogy, Mineralogy, Inorganic chemistry, Materials chemistry, Surface chemistry, Chemistry, Nanoscience and technology, Nanoscale materials, Materials science, Nanoscale materials

## Abstract

Morphology-controlled strontianite nanostructures have attracted interest in various fields, such as electrocatalyst and photocatalysts. Basic additives in aqueous strontium solutions is commonly used in controlling strontianite nanostructures. Here, we show that trace water also serves an important role in forming and structuring vertically oriented strontianite nanorod arrays on strontium compounds. Using in situ Raman spectroscopy, we monitored the structural evolution from hydrated strontium to strontianite nanorods, demonstrating the epitaxial growth by vapor–liquid–solid mechanism. Water molecules cause not only the exsolution of Sr liquid droplets on the surface but also the uptake of airborne CO_2_ followed by its ionization to CO_3_^2−^. The existence of intermediate SrHO^+^–OCO_2_^2−^ phase indicates the interaction of CO_3_^2−^ with SrOH^+^ in Sr(OH)_x_(H_2_O)_y_ cluster to orient strontianite crystals. X-ray diffraction simulation and transmission electron microscopy identify the preferred-orientation plane of the 1D nanostructures as the (002) plane, i.e., the growth along the c-axis. The anisotropic growth habit is found to be affected by the kinetics of carbonation. This study paves the way for designing and developing 1D architecture of alkaline earth metal carbonates by a simple method without external additives at room temperature.

## Introduction

Strontium is an alkaline earth metal that has two electrons in the outer valence shell. It has a very low electronegativity (1.0), thus tending to be readily ionized as Sr^2+^ to react with oxygen and H_2_O in air^1^. It is an important element as A-site dopant or ingredient in perovskite-type electrodes and catalysts^2–4^. For instance, in (La,Sr)MnO_3_ and (La,Sr)(Co,Fe)O_3_ perovskite cathodes, the Sr^2+^ substitution for La^3+^ provides a structure that is favorable to polaron hopping, improving the electrical conductivity in solid oxide fuel cells (SOFC)^5–8^.

Sr surface segregation frequently occurs in perovskite structures and is among important issues since the near-surface structure determines the overall properties of the materials. The relatively large size of Sr^2+^ (0.144 nm for 12 coordination) is regarded as the cause of the segregation^9–14^. In humid environments, the segregation is accelerated as the absorption of H_2_O distorts the lattice, allowing for facile migration of Sr ions toward the surface^15–17^. The segregated Sr is likely to exist as SrCO_3_ on the surface^18^.

Strontianite, or SrCO_3_, has a wide range of applications, such as in dye-sensitized solar cell^19^, thermochemical energy storage^20^, cataluminescence-based sensor^21^, eletrocatalyst^22^, and photocatalyst^23–25^, apart from raw material in industry. The morphology control of SrCO_3_ has attracted considerable interest as providing the opportunity to explore and develop novel properties: e.g., urchin-like SrCO_3_ particles showed enhanced specific capacitance^26^, and vertically-oriented SrCO_3_ nanorods exhibited photoluminescence (PL) quenching over the full solar spectrum range^27^.

In fabricating SrCO_3_ hierarchical superstructures (e.g., needle-, dumbbell-, and flower-like morphologies), various synthesis methods including hydrothermal route have been developed, where strontium nitrate, chloride, and acetate are frequently used as the Sr source (listed in Supplementary Table S1 online)^26–42^. Our recent work showed that the continuous Sr segregation induced by H_2_O absorption leads to the formation of SrCO_3_ (Supplementary Fig. S1 online), especially with nanorod morphology^16^. However, no report has been found for the SrCO_3_ formation via the H_2_O absorption induced Sr-segregation in ambient environment, which could be closely related to the biomineral carbonation in nature. Despite great advances in the synthesis and application, studies on the fundamental science of SrCO_3_ formation have lagged behind. For further design and development of one-dimensional (1D) architectures of SrCO_3_, the growth mechanism of SrCO_3_ nanorods needs to be understood.

The current study focuses on the structuring role of water in the growth of single-crystalline SrCO_3_ nanorod arrays. The evolution of hydrated strontium into SrCO_3_ is investigated by in situ Raman spectroscopy. It is found that the hydrated Sr has a strong tendency to absorb CO_2_ in air whereas non-hydrated Sr does not. The observation of the intermediate phase, as well as thermodynamic considerations, elucidates the reaction pathway for the formation of SrCO_3_. X-ray diffraction (XRD) simulation and transmission electron microscopy (TEM) analyses further reveal that the epitaxial growth of SrCO_3_ occurs along the *c*-axis, leaving stacking faults behind.

## Results

### SrCO_3_ vertical growth on hydrated strontium compounds

**Figure 1:**
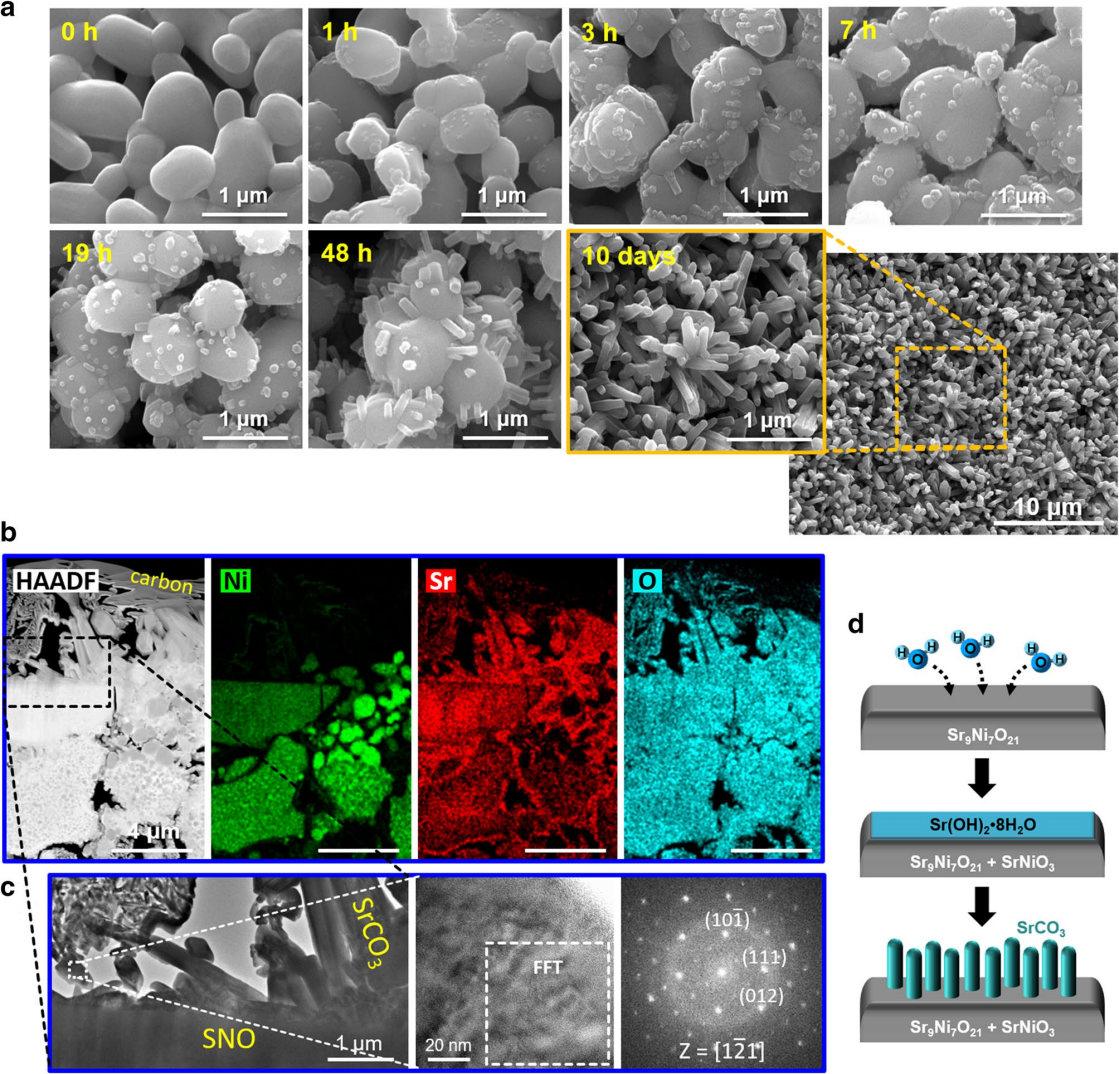
(**a**) SEM images of the surface of strontium nickel oxide pellet before and after the exposure to a humid environment (2.7% H_2_O content) for 1 h, 3 h, 7 h, 19 h, 48 h, and 10 days. The SEM–EDS spectrum of the nanorod array is also presented in Supplementary Fig. S2 online. It is noted that these samples were dried in ambient air (0.3% H_2_O content) for 2 h after exposure to the humid environment. SEM images of the samples that were not dried are also displayed in Supplementary Fig. S3 online. (**b**) High-angle annular dark-field (HAADF) and elemental mapping images (Ni, Sr, and O) of the cross-section of the SNO pellet exposed to the humid environment for 5 days, analyzed using FIB and STEM-EDS. (**c**) High-resolution TEM images of the nanorod grown on the SNO surface and the corresponding FFT pattern indexed in the *Pmcn* space group of SrCO_3_. (**d**) Schematic representation of the structural evolution of SNO by hydration and the accompanying SrCO_3_ nanorod growth.

As an example, Sr_9_Ni_7_O_21_, which is one of Sr-enriched structures and subject to Sr segregation in humid environment, was used to investigate the growth of SrCO_3_ nanorods from hydrated strontium compounds. Figure 1a shows scanning electron microscopy (SEM) images of the surface of strontium nickel oxide (SNO; Sr_9_Ni_7_O_21_) exposed to a humid environment (2.7% H_2_O) for 0–48 h and 10 days. It is seen that Sr is readily segregated from SNO by absorbing moisture, leading to the growth of nanorods on the surface. Figure 1b displays the cross-sectional images (HAADF and elemental mapping) of the nanorods on the SNO surface. Sr-enrichment is observed in the surface nanorods and along the SNO grain boundaries. The nanorod is identified as SrCO_3_ (*Pmcn*; JCPDS No. 05-0418) by the fast-Fourier-transform (FFT) technique (Fig. 1c), thus demonstrating Sr-segregation and SrCO_3_ growth from a Sr-rich compound. It should be noted that the strontium, segregated by H_2_O absorption, would exist initially as Sr(OH)_2_·8H_2_O, according to our previous study^16^. The hydrated Sr-hydroxide is subject to reacting with CO_2_ in air, resulting in SrCO_3_, as per the reaction.

1 $${\text{Sr}}({\text{OH}})_{2} \cdot 8{\text{H}}_{2} {\text{O }} + {\text{ CO}}_{{2{\text{(g)}}}} \to {\text{ SrCO}}_{3} + \, 9{\text{H}}_{2} {\text{O}}$$

It is interesting that the SrCO_3_ here grows in one-dimension, eventually forming SrCO_3_ nanorod arrays on the SNO surface (Fig. 1d). Such a phenomenon may occur in other alkaline-earth-metal compounds as well. For instance, SrCO_3_ nanowhiskers were found to grow from Sr_4_Mn_3_O_10_ in humid environment (see Supplementary Fig. S4 online), which have potential applications as Cr/S getters^43^ in high-temperature electrochemical systems. While water molecules are considered contributors to the directional growth, the role of water molecules in Sr carbonation is rarely studied. It remains to be discovered how Sr(OH)_2_·8H_2_O in air transforms into SrCO_3_ and how the resultant SrCO_3_ has 1D morphology. These questions will be answered in the following sections.

### Effect of moisture on SrCO_3_ formation

In order to study the influence of moisture on the formation of SrCO_3_, as-received SrO and Sr(OH)_2_·8H_2_O powders were placed for 24 h in an ambient room air (1.2% H_2_O present; and 400 ppm CO_2_) or under a flow of CO_2_–air mixed gas (H_2_O absent; and 30% CO_2_). Their morphology and structure changes were analyzed by SEM and XRD.

**Figure 2:**
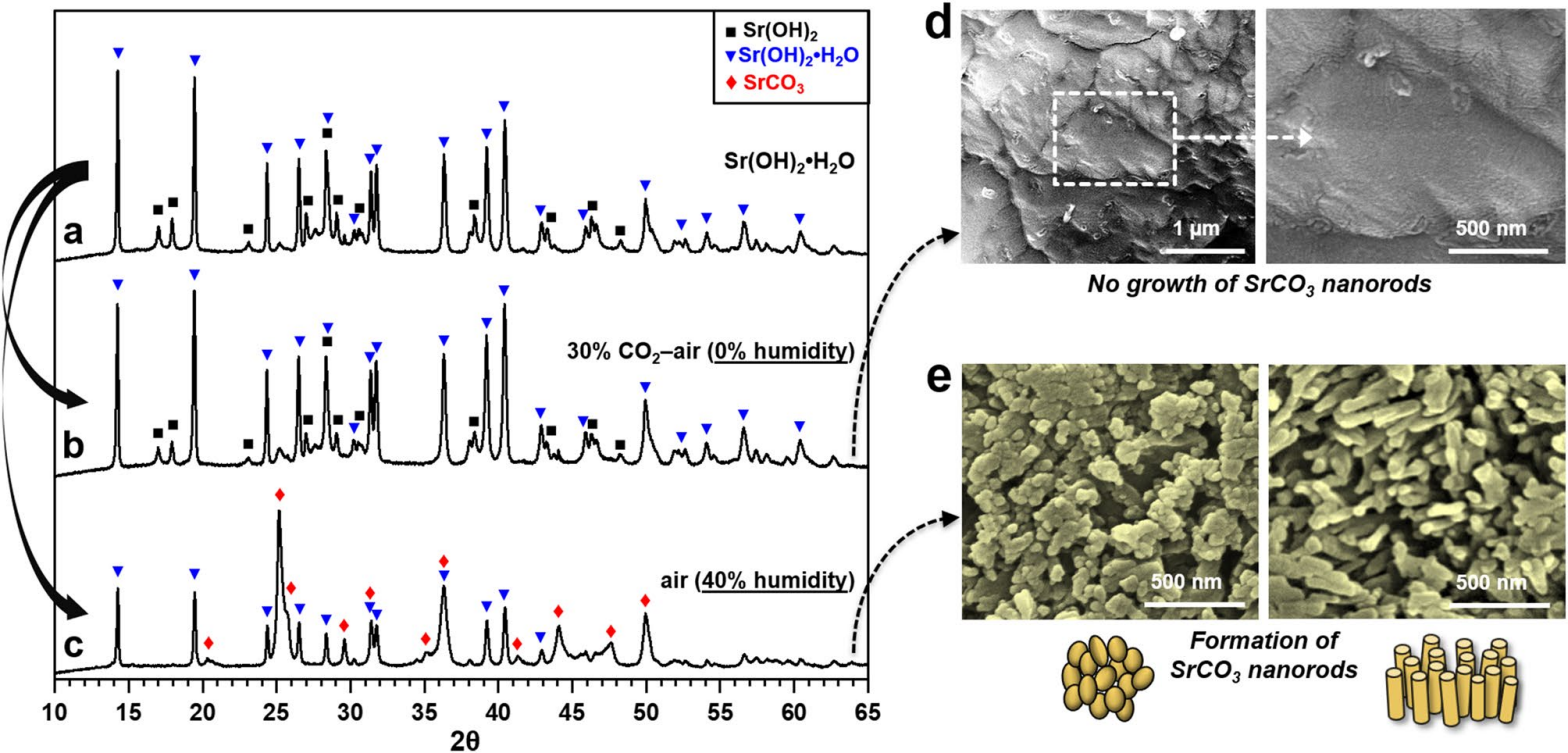
XRD patterns of SrO before and after exposure to ambient room air (1.2% H_2_O present, and 400 ppm CO_2_) and a CO_2_-air mixed gas (H_2_O absent, and 30% CO_2_) for 24 h (**a**–**c**), and their corresponding SEM images (**d**,**e**). Note that the XRD pattern of SrO could not be properly recorded because SrO was readily hydrated by absorbing airborne moisture in the middle of XRD analysis; thus, Sr(OH)_2_ and Sr(OH)_2_·H_2_O were detected instead of SrO.

**Figure 3:**
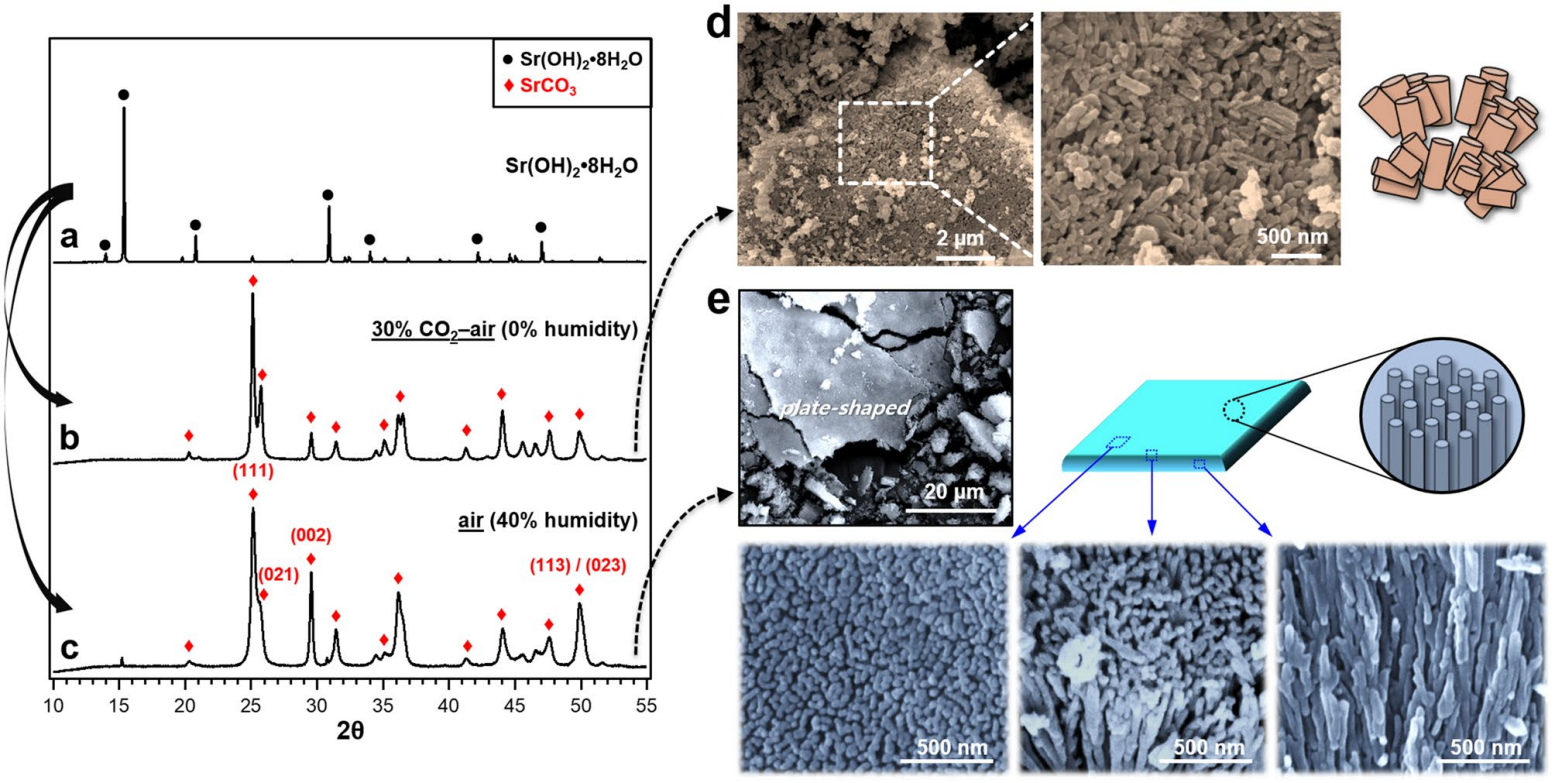
XRD patterns of Sr(OH)_2_·8H_2_O before and after exposure to an ambient room air (1.2% H_2_O present, and 400 ppm CO_2_) and a CO_2_-air mixed gas (H_2_O absent, and 30% CO_2_) for 24 h (**a**–**c**), and their corresponding SEM images (**d**,**e**).

Figure 2 displays XRD patterns and SEM images of the SrO exposed to the two different atmospheric conditions. Several distinguishing characteristics of the reactivity of SrO with air were revealed as follows. First, as-received SrO (Alfa Aesar, USA) was identified as Sr(OH)_2_·H_2_O with a small portion of Sr(OH)_2_ by XRD analysis (Fig. 2a) since SrO was readily hydrated by absorbing airborne moisture during the XRD analysis, indicating the hygroscopic nature of SrO. Second, it is interesting that SrO (and if any existing Sr(OH)_2_) does not react with airborne CO_2_ in the absence of moisture; that is, no SrCO_3_ formed under dry CO_2_–air flow, albeit containing a high concentration of CO_2_ (30%) (Fig. 2b,d). In other words, the reaction between SrO and CO_2_ occurs only in the presence of moisture; indeed, SrCO_3_ was produced in an ambient air containing moisture (1.2% H_2_O in this case) although the concentration of CO_2_ was as low as 400 ppm (Fig. 2c). Third, a morphological transformation into nanorods during the SrCO_3_ formation was observed (Fig. 2e). Further information to understand the anisotropic growth of SrCO_3_ is given in Fig. 3.

Figure 3 shows XRD patterns and SEM images of the Sr(OH)_2_·8H_2_O exposed to the two different atmospheric conditions. It is found that the exposure of hydrated strontium (i.e., Sr(OH)_2_·8H_2_O) to CO_2_, regardless of the presence of moisture, results in the formation of SrCO_3_ (Fig. 3a–c), corresponding to the above discussion. SEM observations reveal that the SrCO_3_ tends to grow in one-dimension when derived from Sr(OH)_2_·8H_2_O (Fig. 3d,e). This tendency is very prominent for the Sr(OH)_2_·8H_2_O exposed to air containing moisture (Fig. 3e). That is, quasi-vertically aligned SrCO_3_ nanorod arrays were produced from the Sr(OH)_2_·8H_2_O further hydrated in the presence of moisture (i.e., 1.2% H_2_O with 400 ppm CO_2_ in an ambient air) (Fig. 3e) whereas irregularly aligned SrCO_3_ nanorods with relatively small aspect ratios grew in the moisture-free environment (i.e., dry air with 30% CO_2_) (Fig. 3d).

The anisotropic growth of SrCO_3_ from Sr(OH)_2_·8H_2_O in air can be explained by considering the reaction kinetics. As per Eq. (1), in the very low partial pressure of CO_2_ (i.e., 400 ppm in air), the reaction rate for SrCO_3_ formation would be very slow, so that the clusters and nuclei of SrCO_3_ afford movement and rotation to assemble along their preferential orientation, resulting in the 1D-oriented growth of SrCO_3_. This is possible because moisture can hydrate the surface strontium and form an aqueous liquid layer over the solid crystalline Sr(OH)_2_·8H_2_O where SrCO_3_ nuclei can move and rotate freely; indeed, the presence of aqueous liquid layer on SNO surface was found (see Supplementary Fig. S3 online).

### Preferred growth orientation of SrCO_3_

**Figure 4:**
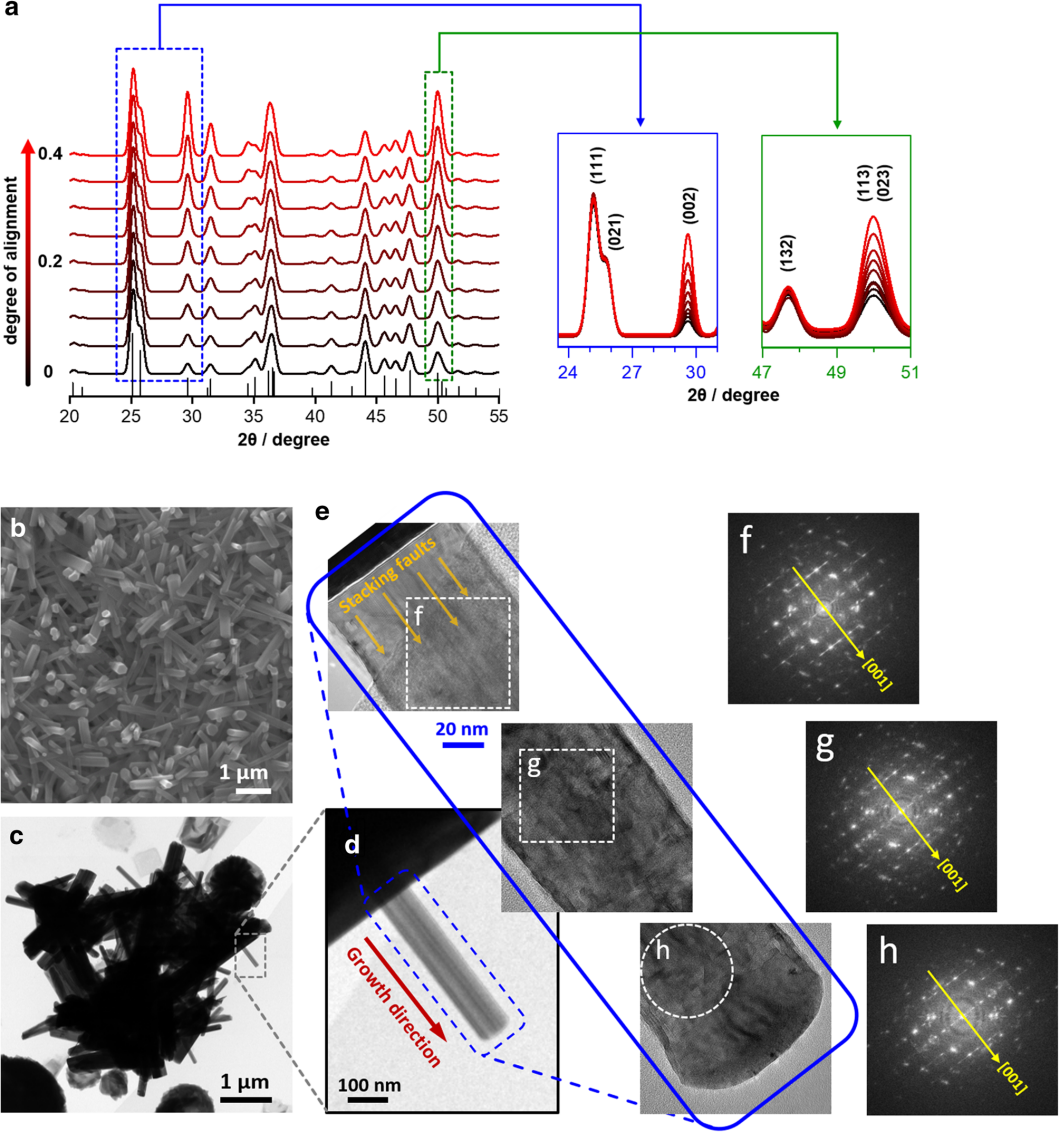
(**a**) Simulated XRD patterns of the SrCO_3_ (*Pmcn*; orthorhombic; and D = 14 nm) with the preferred orientation along (002) plane (i.e., *c*-axis) as a function of the degree of alignment. The degree of alignment is represented by a fraction, with 0 indicating a completely random-oriented structure, and 1 being a fully oriented structure (e.g., precisely parallelly aligned rods). (**b**) SEM and (**c**) TEM images of SrCO_3_ nanorods. (**d**,**e**) Low- and high-magnification TEM images of a single-crystalline SrCO_3_ nanorod. (**f**–**h**) FFT diffraction patterns of selected areas in (**e**), indexed to the orthorhombic SrCO_3_ (*Pmcn*; JCPDS No. 05-0418) (Supplementary Fig. S7 online).

The preferred orientation for the growth of SrCO_3_ is further investigated by XRD simulations together with the measured XRD data. First, the XRD pattern of the quasi-vertically aligned SrCO_3_ nanorod arrays in Fig. 3c was closely inspected. The intensity of (111) peak is the strongest while that of (021) peak seems to be weakened, compared with Fig. 3b. This has often misled us to make an incorrect conclusion that the SrCO_3_ crystal grows mainly along the (111) face^41,44^. However, such XRD pattern (at ~ 25.5° in Fig. 3c) is likely due to the broadening and overlapping of (111) and (021) peaks, rather than to the (111) oriented growth, which is in good agreement with the XRD profile simulated as a function of SrCO_3_ crystallite sizes (see Supplementary Fig. S5 online). Since the peaks for (002), (113) and (023) planes, as shown in Fig. 3c, have much stronger intensities than those of typical SrCO_3_ (Supplementary Fig. S6 online), those planes can be nominated as the preferred orientation of the SrCO_3_ growth. Next, XRD simulations on the planes were performed using CrystalDiffract software to identify the preferred orientation plane. Figure 4a displays simulated XRD patterns of the SrCO_3_ with (002) preferred orientation as a function of the degree of alignment. It is found that the intensity of (113)/(023) peak increases, along with (002) peak, as shown in the XRD patterns of the SrCO_3_ with preferred orientation in (002) plane (Fig. 4a: right insets). This simulation result is well matched to the measured one in Fig. 3c, suggesting that the SrCO_3_ nanorod grows preferentially at $\langle {001} \rangle$ direction (i.e., along *c*-axis).

TEM analysis was performed to further validate the above results. Figure 4b,c show SEM and TEM images of nanorods grown from Sr(OH)_2_·8H_2_O in humid condition. The low- and high-resolution (HR) TEM images of a single nanorod in Fig. 4d,e show fringes parallel to the growth direction of the nanorod (marked by arrows), indicative of planar defects. The FFT patterns of selected areas in Fig. 4e are displayed in Fig. 4f–h. The diffraction spots are indexed to the orthorhombic SrCO_3_ (*Pmcn*; JCPDS No. 05-0418) where the streaks, lying parallel to the [010] direction, reflect local disorder of the atomic stacking (i.e., stacking faults) as being perpendicular to the fringes in the HR-TEM image (see Supplementary Fig. S7 online). The distance between the streaks, normal to the growth direction, corresponds to the (001) plane, i.e., 0.60 nm, demonstrating the growth direction of the nanorod to be $\langle {001} \rangle$. Note that this kind of pattern appears in all regions of the nanorod (Fig. 4f–h). Hence, it is concluded that the SrCO_3_ nuclei tend to assemble along the *c*-axis leaving stacking faults behind, which is in good agreement with the result from XRD simulation.

### Chemical structure evolution of hydrated SrO to SrCO_3_

**Figure 5:**
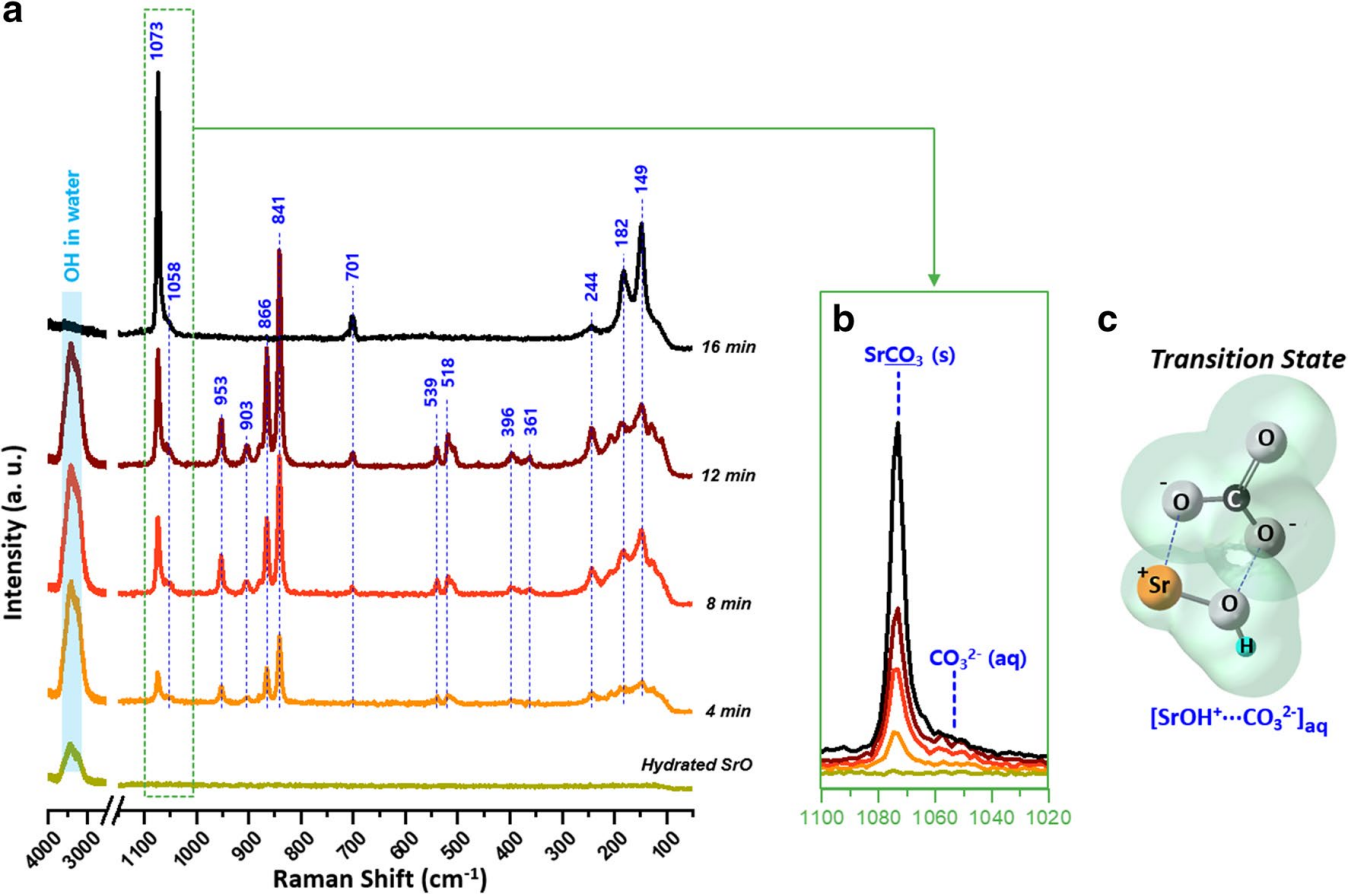
(**a**) Raman spectra of hydrated SrO in ambient air, recorded over time (0, 4, 8, 12, and 16 min). (**b**) Superimposed Raman spectra at the range of 1020–1100 cm^−1^. (**c**) A structure of the transition-state intermediate, SrOH^+^⋯CO_3_^2−^. Vibration modes of the Raman spectra are displayed in Supplementary Table S2.

The reaction pathway for the carbonation of hydrated SrO was investigated by in situ Raman spectroscopy. Figure 5 shows representative Raman spectra of hydrated SrO that is exposed to ambient air for up to 16 min. Vibration modes of the Raman spectra are displayed in Supplementary Table S2, according to literature^45–62^.

The initial Raman spectrum of SrO soaked with water (Fig. 5a: Hydrated SrO) is dominated by a broad band at 3000–3700 cm^−1^ owing to the stretching of O–H in water^46^, implying hydration of SrO. Over time, the peaks ascribable to SrCO_3_ (149, 182, 244, 701, and 1073 cm^−1^) rise, indicating the carbonation of hydrated strontium (Fig. 5a: 4–16 min). Careful observations on the peak for SrCO_3_ at 1020–1100 cm^−1^ (Fig. 5b) reveal that the growth of the SrCO_3_ peak is accompanied by the rise of the small band at ~ 1055 cm^−1^ (assigned to CO_3_^2−^ ions in aqueous solution^51,52^) which is not present in the normal spectrum of solid SrCO_3_ (see Supplementary Fig. S8 online). The results demonstrate that (i) hydrated strontium has a high tendency to absorb CO_2_ and (ii) the absorbed CO_2_ is present as CO_3_^2−^ ions which are supposed to react with strontium to form SrCO_3_. These results are supported by the facts that aqueous alkaline-earth-metal hydroxide solutions are strong bases (e.g., pH = 11.27–13.09 at 1–100 mM Sr(OH)_2_) and that the basic solution has the high solubility of CO_2_ (supposed to convert to CO_3_^2−^) thus capturing airborne CO_2_ so as to meet the equilibrium condition. For details, see Supplementary Fig. S9 online.

While the dissolved carbon is present as CO_3_^2−^ in the basic aqueous solution, the chemical structure of strontium has not been completely determined so far; for instance, the Sr^2+^ coordination number surrounded by H_2_O/OH^−^ was variously suggested to be six, or eight, etc.^63–73^. Aside from such discrepancy, there is a general consensus that hydrated Sr^2+^ clusters have flexible structures freely exchanging H_2_O and OH^−^ between the first and second shells^74,75^. This indicates that Sr^2+^ hydroxide clusters with Sr^2+^–OH^−^ interaction (mostly mono/di-hydroxides^75^) are always present in high pH aqueous solutions (OH^−^ abundant); however, Sr^2+^ and OH^−^ are not directly connected in both Sr(OH)_2_·8H_2_O and Sr(H_2_O)_7_(OH)^+^ solid phases (instead, only Sr^2+^–H_2_O interaction exists unless further hydrated)^69,76^.

**Figure 6:**
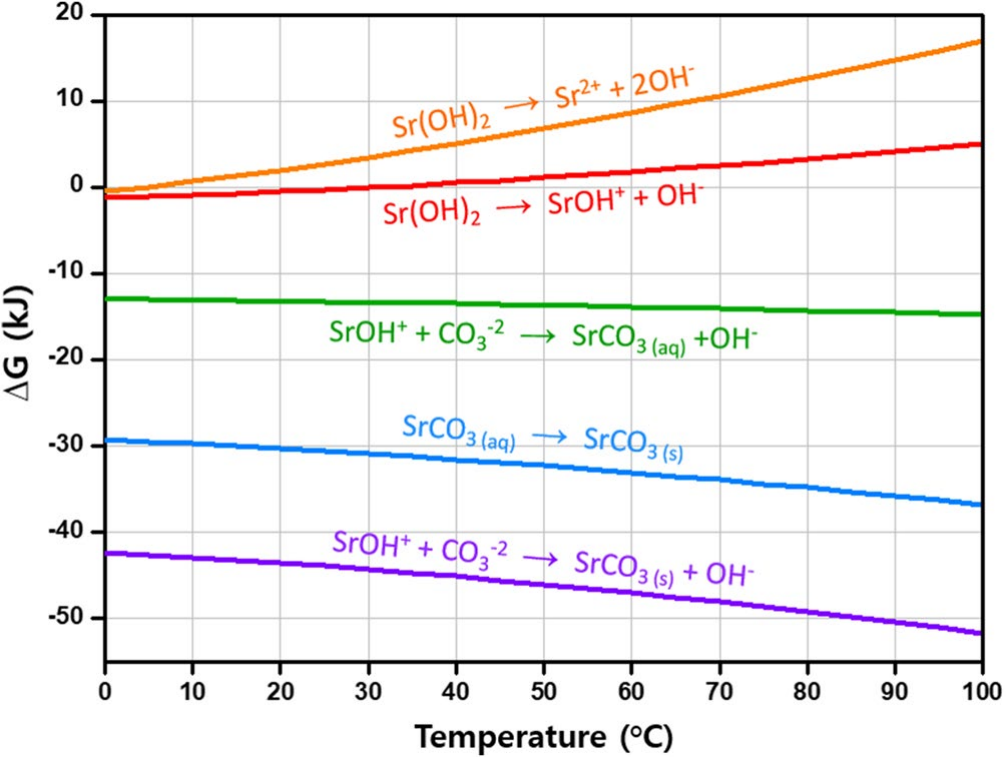
Gibbs free energy changes for the plausible reactions of strontium hydroxide and carbon ions in aqueous solution at 0–100 °C, plotted using HSC Chemistry software.

This assumption is thermodynamically reasonable. Figure 6 displays Gibbs free energies for the plausible reactions of strontium hydroxide and carbon ions in aqueous solution. Based on the Gibbs free energy changes of the formation of SrOH^+^ and Sr^2+^ ions (red and orange curves in Fig. 6, respectively), it is energetically favorable for Sr^2+^ to be paired with OH^−^, namely SrOH^+^, which corresponds to the computational study^64,77^. After many attempts, SrOH^+^ was indeed observed by in situ Raman spectroscopy. The signals attributable to SrOH^+^ (bands at 361, 396, 518, and 539 cm^−1^) and to O–O stretching in Sr(O_2_)_1−x_–O_x_ (bands at 840–860 cm^−1^) were detected (Fig. 5a: 4 min). The intensity of the bands relating to SrOH^+^, Sr(O_2_)_1−x_–O_x_, and SrCO_3_ increased simultaneously during the Sr-carbonation (Fig. 5a: 4–12 min). After completion of the carbonation (Fig. 5a: 16 min), the bands disappeared, leaving only those for SrCO_3_. These findings indicate that SrOH^+^–CO_3_^2−^, or SrHO^+^–OCO_2_^2−^ (depicted in Fig. 5c), is an intermediate phase during the carbonation of hydrated strontium. It is thus evident that, in the Sr liquid droplets, the interaction of CO_3_^2−^ with SrOH^+^ in Sr(OH)_x_(H_2_O)_y_ cluster orients the SrCO_3_ nuclei, and subsequent dehydration leaves SrCO_3_ nanorods.

## Discussion

We have studied water-mediated Sr-precipitate and growth of strontianite nanorod arrays on the surface of strontium compounds in ambient conditions. As a case study, we choose to investigate Sr_9_Ni_7_O_21_, which is a Cr gettering material^78^. The compound is prone to Sr-segregation in air by absorbing airborne moisture and transforming the structure as per the reaction: Sr_9_Ni_7_O_21_ + nH_2_O → 7SrNiO_3_ + 2Sr(OH)_2_·8H_2_O. The segregated Sr(OH)_2_·8H_2_O subsequently converts to SrCO_3_ in ambient air, which interestingly has nanorod morphology. Time-dependent SEM analysis demonstrated that the growth of SrCO_3_ occurs in one direction when derived from hydrated SrO, such as Sr(OH)_2_·8H_2_O.

The effect of water molecules on the formation and growth of SrCO_3_ during the carbonation has been elucidated. In the absence of water molecules, SrO does not react with CO_2_ even under high CO_2_ partial pressure (30%), whereas SrO readily reacts with CO_2_ in ambient air (water molecule present) despite its low concentration (400 ppm). These results suggest that the carbonation of SrO at ordinary temperatures occurs via hydration in the presence of moisture, as illustrated in Supplementary Fig. S10 online. The driving force for the Sr-carbonation can be understood from the perspective of CO_2_ dissolution in aqueous alkaline solution. Basically, aqueous alkaline solutions have high solubility of carbonate ions due to high pH (i.e., high concentration of OH^−^). Likewise, the moisture absorption endows strontium with more alkaline character, and thus the hydrated strontium, such as Sr(OH)_2_·8H_2_O and Sr(OH)_2_·xH_2_O, becomes capable of absorbing CO_2_ in air. In the alkaline condition of hydrated strontium, Sr^2+^ is likely to be coupled with OH^−^ (i.e., SrOH^+^), and the absorbed CO_2_ converts into CO_3_^2−^. Subsequent interaction of the Sr and C ions leads to the formation of SrCO_3_ nuclei (Supplementary Fig. S10 online). Hence, the generally known fact “SrO is likely to react with CO_2_” can be better understood as “Once hydrated, SrO reacts with CO_2_”.

It is also found that the morphology of the resulting SrCO_3_ is affected by the kinetics of SrCO_3_ formation. The Sr-carbonation via hydration is known to occur following the reaction, Sr(OH)_2_·8H_2_O + CO_2_ → SrCO_3_ + 9H_2_O. It is thus implied that the kinetics of SrCO_3_ formation is slower under low CO_2_ and high H_2_O partial pressures (or humidity level). Experimental results in Figs. 2 and 3 showed the growth trend of SrCO_3_ under different atmospheric conditions. When SrCO_3_ forms under high *p*CO_2_ and low *p*H_2_O (i.e., favorable for fast reaction kinetics), it has spherical morphology, suggesting the isotropic growth of SrCO_3_. In contrast, when SrCO_3_ forms under low *p*CO_2_ and high *p*H_2_O (i.e., desirable for slow reaction kinetics), it is likely to have nanorod morphology, indicating the anisotropic growth of SrCO_3_. That is, the slower the reaction kinetics, the more the SrCO_3_ grows in one direction (Supplementary Fig. S11 online). Thus, it is considered that, when sufficient time is given for precursor ions to move and rotate, anisotropic assembly occurs particularly along [001] direction resulting in *c*-axis oriented SrCO_3_ nanorods.

**Figure 7:**
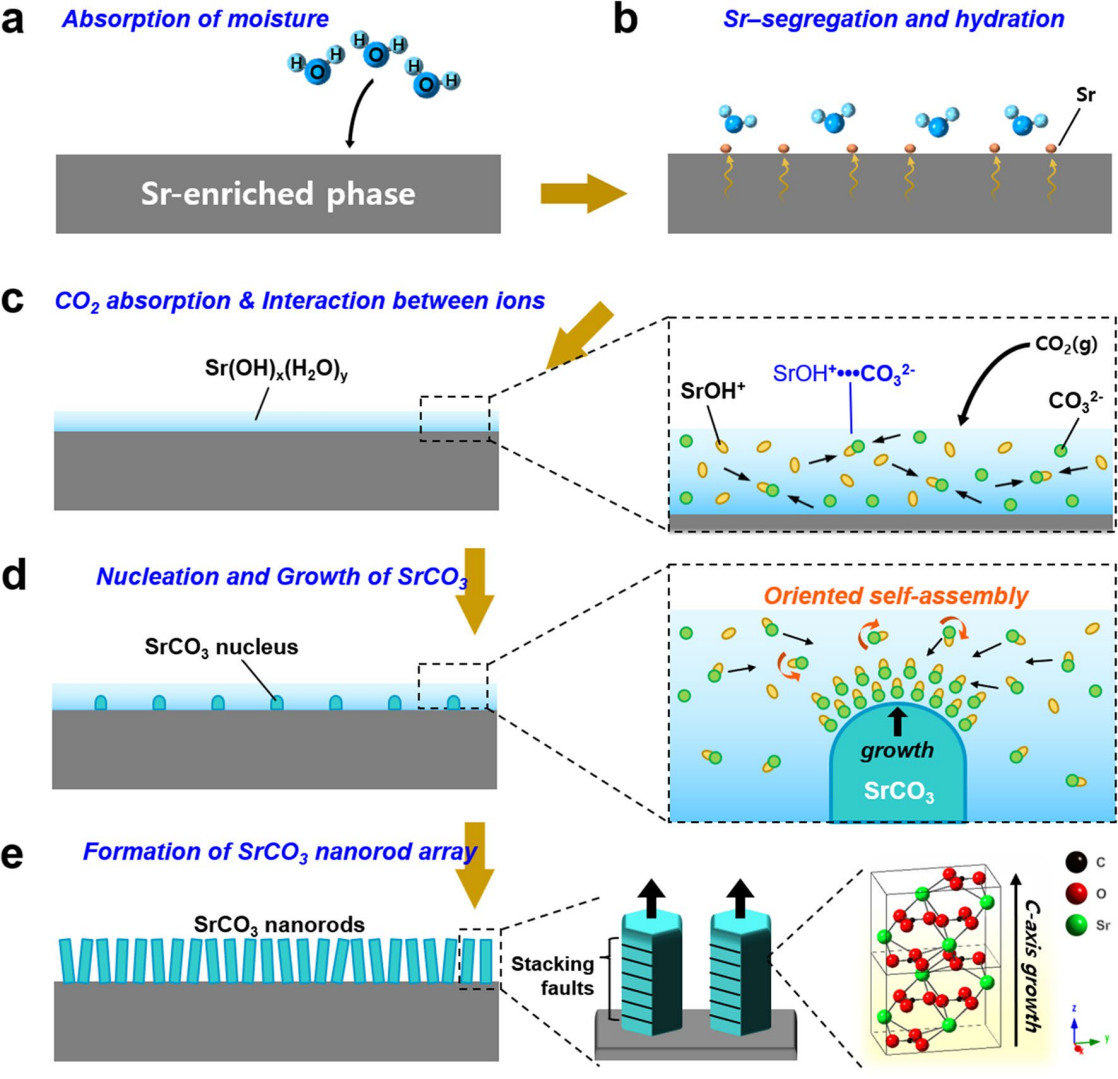
Schematic diagram for the process of the growth of SrCO_3_ nanorod arrays from hydrated strontium that is segregated from Sr-containing substrates.

The formation process of SrCO_3_ nanorod arrays from Sr-enriched phases by vapor–liquid–solid mechanism in ambient conditions is summarized in Fig. 7. First, the Sr-terminated surface, being hygroscopic, attracts and absorbs water molecules in air (Fig. 7a). This leads to the hydration and segregation of strontium onto the surface, thus forming Sr(OH)_2_·H_2_O, Sr(OH)_2_·8H_2_O, and Sr(OH)_x_·(H_2_O)_y_ layers (Fig. 7b). The alkaline nature of the hydrated Sr-hydroxide layer causes the uptake of CO_2_ in air, which subsequently converts to HCO_3_^−^ and CO_3_^2−^ (Fig. 7c). In the moistened medium, Sr and C ion complexes, including SrOH^+^ and CO_3_^2−^, freely move, rotate, and interact with each other, leading to the assembly, nucleation, and growth of SrCO_3_ (Fig. 7d). Particularly, the growth occurs preferentially along the *c*-axis, leaving staking faults behind. In this way, quasi-vertically aligned SrCO_3_ nanorod arrays with (002) orientation can form at room temperature in ambient air without external addition of carbon source (Fig. 7e). This study paves the way for designing and developing 1D architecture of SrCO_3_ via H_2_O-mediated precipitation from Sr-rich compounds. This strategy can also be applicable to other alkaline-earth-metal compounds to fabricate 1D nanostructures.

## Methods

For the preparation of strontium nickel oxide (Sr_9_Ni_7_O_21_), the mixtures of Sr(OH)_2_·8H_2_O (Sigma-Aldrich, USA) and Ni(OH)_2_ (Sigma-Aldrich, USA) were heat-treated at 850–900 °C in air, where the molar ratio of Sr to Ni was 35:65 (see Ref.^14^). As-prepared SNO powder was uniaxially pressed into cylindrical pellets, followed by sintering at 900 °C. The SNO pellets were placed in a humid environment (i.e., a sealed container containing water at the bottom, 2.7% H_2_O content measured by a ThermoPro hygrometer). The pellet surface, which was exposed to the humid environment for 0–48 h and 10 days, was examined by a field-emission environmental scanning electron microscope (FE-ESEM; Quanta 250 FEG, FEI, USA) equipped with an energy dispersive X-ray spectrometer (EDS), for which the specimens were coated with gold either after being dried for 2 h in ambient air or immediately after being taken out from the humid condition. For scanning transmission electron microscopy (STEM) analysis, the SNO pellet, exposed to the humid environment for 5 days, was sliced using a focused ion beam (FIB; Helios Nanolab 460F1, FEI, USA). The specimen was analyzed using STEM (Talos F200X S/TEM, FEI, USA).

The structures and morphologies of as-received SrO (Alfa Aesar, USA) and Sr(OH)_2_·8H_2_O (Sigma-Aldrich, USA), exposed either to an ambient atmosphere (with 400 ppm CO_2_ and 1.2% H_2_O contents) or to CO_2_-air mixture (with 30% CO_2_ and ~ 0% H_2_O contents) at room temperature, were analyzed using SEM and X-ray diffractometer (XRD; D8 Advance, Bruker, Germany) with Cu-kα radiation (λ = 0.1542 nm). CrystalDiffract software (Version 6.8.2, CrystalMaker Software Ltd.) was used to identify and simulate XRD patterns of SrCO_3_ nanorods with different growth directions. The following parameters were used in the simulation: *Pmcn* (orthorhombic); lattice constants a = 5.107 Å, b = 8.414 Å, and c = 6.029 Å; and crystallite size = 14 nm. TEM analysis (Talos F200X S/TEM; FEI, USA) was performed on SrCO_3_ nanorods grown from Sr(OH)_2_·8H_2_O in humid condition. For TEM sample preparation, the nanorods were dispersed in ethanol and dropped onto a carbon-coated grid. TEM images were obtained at an accelerating voltage of 200 kV, followed by fast-Fourier-transformation analysis.

For in situ monitoring of the growth of SrCO_3_ from hydrated SrO, Raman spectroscopy (Ramanscope 2000, Renishaw, Gloucestershire, UK) was employed. As-received SrO particles were drenched in a droplet of DI water on a microscope glass slide to form an aqueous film of strontium hydroxide hydrate. Raman spectra of the film were then recorded over time with a laser of 514.5 nm wavelength. Gibbs free energy changes for the probable reactions between strontium and carbon ions at 0–100 °C were calculated from the database in HSC Chemistry 6 (Outotec, Finland).

## Supplementary Information

Supplementary Information.
