# Supplementary material for: Water mediated growth of oriented single crystalline SrCO3 nanorod arrays on strontium compounds
Source: Sci Rep. 2021 Feb 9;11:3368. doi: 10.1038/s41598-021-82651-0 (PMC7873059; doi:10.1038/s41598-021-82651-0)
Supplement: Supplementary file 1 — Supplementary Information. [file 41598_2021_82651_MOESM1_ESM.pdf]

Supplementary information

Water Mediated Growth of Oriented Single Crystalline $SrCO_3$ Nanorod Arrays on Strontium Compounds

Junsung Hong,[1,2] Su Jeong Heo,[1,3] and Prabhakar Singh[1]*

[1]Department of Materials Science and Engineering, University of Connecticut, Storrs, Connecticut 06269, United States

[2]Current address: Department of Materials Science and Engineering, Northwestern University, Evanston, Illinois 60208, United States

[3]Current address: Materials Science Center, National Renewable Energy Laboratory, Golden, Colorado 80401, United States

Synthesis methods for $SrCO_3$ hierarchical superstructures

Table S1. Methods and chemicals used for the morphology-controlled synthesis of $SrCO_3$, and their applications

Morphology	Method	Chemical	Application	Ref.
Rod-, shuttle-, peanut-, bouquet-, and sphere-like	Screw capped method	Strontium chloride ($SrCl_2$), ammonium carbonate ($(NH_4)_2CO_3$), and natural gums	-	1
Nanorods	Ultrasonic method	Strontium acetate ($Sr(Ac)_2$, or $Sr(CH_3COO)_2$), and sodium hydroxide (NaOH)	-	2
Bundle-like, dumbbell-like, spherical particles	Precipitation	Strontium nitrate ($Sr(NO_3)_2$), sodium carbonate (Na_2CO_3), and poly-(styrene-alt-maleic acid)	-	3
Flower-like	Hydrothermal synthesis	$Sr(NO_3)_2$, and NaOH	-	4
Nanoneedles	Reverse micelle method	$Sr(NO_3)_2$, n-butylalcohol, cyclohexane ($C_4H_{10}O$), and Na_2CO_3	-	5
Nanoparticles	Microwave-assisted method	$Sr(NO_3)_2$, and NaOH	-	6
Whiskers, nanorods, spherical, ellipsoid-like, nanoneedles	Solvothermal synthesis	$Sr(NO_3)_2$, and Na_2CO_3	-	7
Pine-leaf-like	Solvothermal synthesis	$Sr(NO_3)_2$, and NaOH	-	8
Flower-like	Solvothermal synthesis	$SrCl_2$, and urea	-	9
Dandelion-like, and double-trumpet-like	Aqueous solution route	$SrCl_2$, and NaOH	-	10
Micro-needle	Biological synthesis	$SrCl_2$, and fungus	-	11
Spherical, flower-like and pancake-like	Precipitation	$SrCl_2$ and Na_2CO_3 in alcohol or water solution	-	12
Fiber aggregates	Calcination of strontium acetate	$Sr(Ac)_2$, acetic acid (CH_3CO_2H), and 2-methoxyethanol mixture ($C_3H_8O_2$)	Photoluminescence	13
Urchin-like	Hydrothermal synthesis	$Sr(NO_3)_2$, and urea	Capacitor	14
Microspindle-, nanopompon-, microdumbbell-, prism-, and branch-like	Hydrothermal synthesis	$Sr(NO_3)_2$, and $(NH_4)_2CO_3$	Photocatalytic oxidation of hydrocarbon gases	15
Quasi-vertically grown nanoneedles	Combined electrospinning-calcination technique	$Sr(Ac)_2$, and polyvinyl alcohol	Photoluminescence quenching	16

Observation of strontium carbonation in humid environment

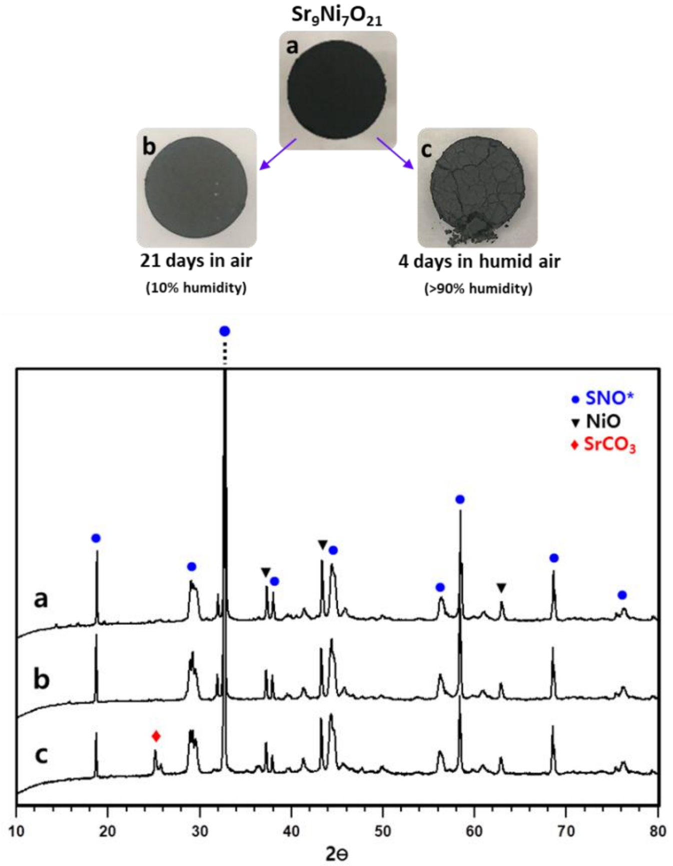

Figure S1. Photos and XRD patterns of (a) as-prepared SNO pellet, (b) SNO pellet exposed to an ambient atmosphere (10% relative humidity) for 21 days at room temperature, and (c) SNO pellet placed in a humid environment (>90% relative humidity) for 4 days. A strontium nickel oxide (SNO) was prepared by the conventional co-precipitation method from strontium and nickel nitrates (11:10 mole ratio) (Sigma-Aldrich, USA).[17,18] As-prepared SNO powder was uniaxially pressed to pellets, followed by sintering in a furnace at 900 °C for 20 h in air. Two SNO pellets were placed in an ambient atmosphere (10% relative humidity) and in a humid environment (>90% relative humidity), respectively. Their structures before and after the placement were analyzed using an X-ray diffractometer (XRD; D8 Advance, Bruker, Germany) with Cu kα radiation (λ = 0.1542 nm). The as-synthesized SNO is mostly composed of $Sr_9Ni_7O_{21}$ phase with a small amount of NiO (Supplementary Figure S1a), and the structure had kept stable for 21 days (Supplementary Figure S1b). In a humid environment, however, the pellet surface had got cracks and been broken only after 4 days, while $SrCO_3$ phase (at 2θ = ~25°) formed (Supplementary Figure S1c), implying the carbonation of segregated Sr in humid environment.

Observation of $SrCO_3$ nanorod arrays

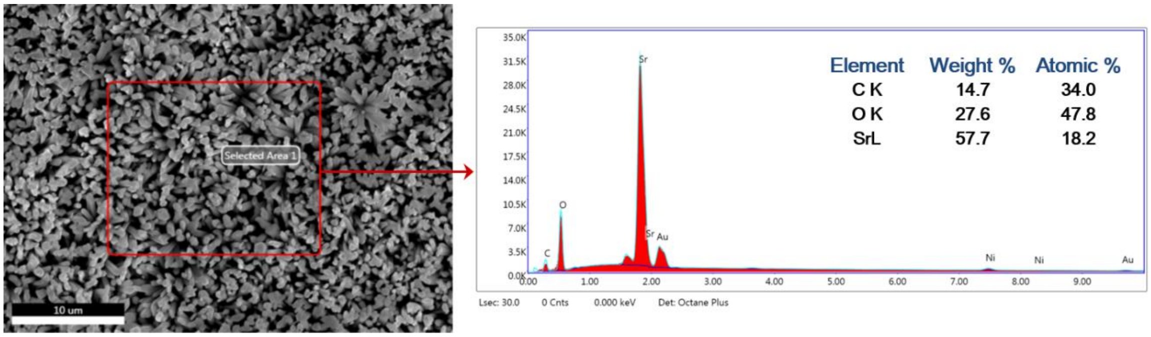

Figure S2. SEM image and EDS spectrum of the nanorod array grown on the surface of SNO pellet after exposure to a humid environment (2.7% H_2O content) for 10 days.

Softened structure of $Sr(OH)_2{\cdot}8H_2O$ by hydration

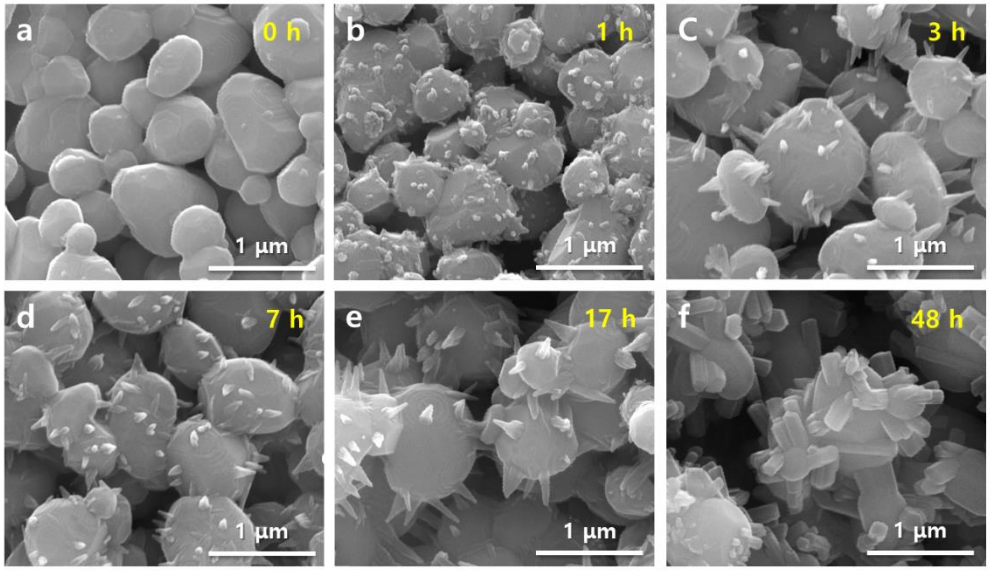

Figure S3. SEM images of the surface of strontium nickel oxide pellet before (a) and after exposure to a humid environment (2.7% H_2O content) for 1 h (b), 3 h (c), 7 h (d), 17 h (e), and 48 h (f). The samples were vacuumed at 50 mTorr immediately after taking out the samples from the humid environment. In humid environment, strontium was segregated as $Sr(OH)_2{\cdot}8H_2O$ onto the SNO surface. Then, the SNO was vacuumed at 50 mTorr. During the vacuum pumping, the $Sr(OH)_2{\cdot}8H_2O$ appeared to be stretched to yield needle-like morphology. It is thus indicated that each particle surface has aqueous liquid $Sr(OH)_x{\cdot}(H_2O)_y$ layer over crystalline $Sr(OH)_2{\cdot}8H_2O$, which facilitates the oriented growth of $SrCO_3$.

Growth of 1D-structural $SrCO_3$ from $Sr_4Mn_3O_{10}$

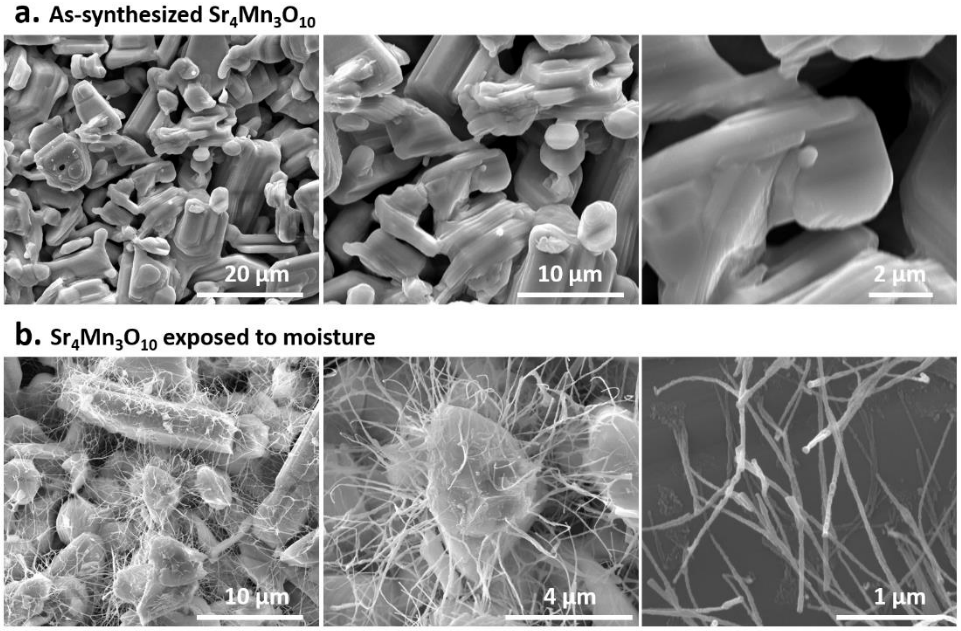

Figure S4. SEM images of (a) as-synthesized $Sr_4Mn_3O_{10}$ and (b) the $Sr_4Mn_3O_{10}$ exposed to a humid environment (3%H_2O–air) for 5 days. In addition to $Sr_9Ni_7O_{21}$, another Sr-enriched compound ($Sr_4Mn_3O_{10}$) was used to verify the growth of 1D-structural $SrCO_3$ via water-mediated Sr-precipitation. The strontium manganese oxide ($Sr_4Mn_3O_{10}$) was synthesized by conventional solid-state reaction of $Sr(OH)_2{\cdot}8H_2O$ and MnO_2 at 1350–1400 °C in air. The as-synthesized $Sr_4Mn_3O_{10}$ pellet was placed in a humid chamber (3%H_2O-air) for 5 days. The $Sr_4Mn_3O_{10}$ pellet before and after exposure to the humid condition was observed using SEM. As shown in Supplementary Figure S4, the surface of $Sr_4Mn_3O_{10}$ particles was covered with $SrCO_3$ nanowhiskers, indicating the precipitation and 1D-vertical growth of $SrCO_3$.

Effect of $SrCO_3$ crystallite size on XRD peak broadening

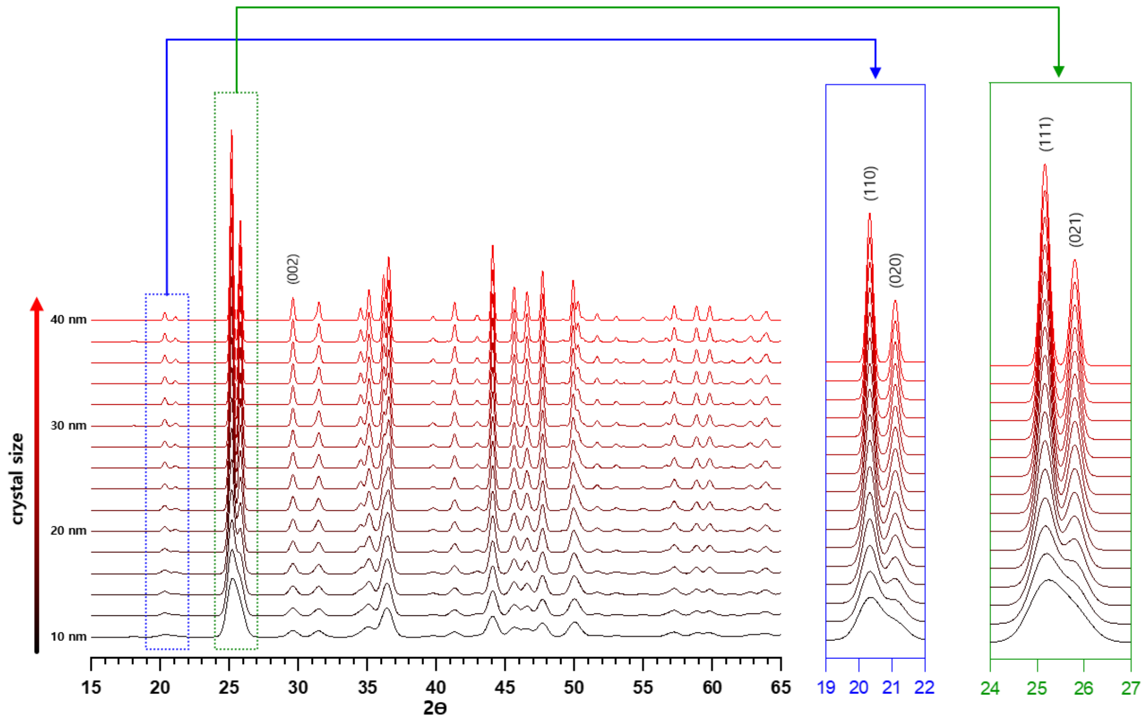

Figure S5. Changes in the XRD patterns of $SrCO_3$ (*Pmcn*; orthorhombic; and a = 5.107 Å, b = 8.414 Å, and c = 6.029 Å) as a function of the crystal size, where the XRD patterns were produced using CrystalDiffract 6.8.2 software (CrystalMaker Software Ltd.)[19]. As the crystallite size of $SrCO_3$ decreases from 40 nm to 10 nm, all diffraction peaks were broadened. In particular, the peaks for (020) and (021) planes (at 2θ = ~21° and ~26°, respectively) became almost disappeared. The simulated XRD patterns were well matched with the experimental result in Supplementary Figure S6.

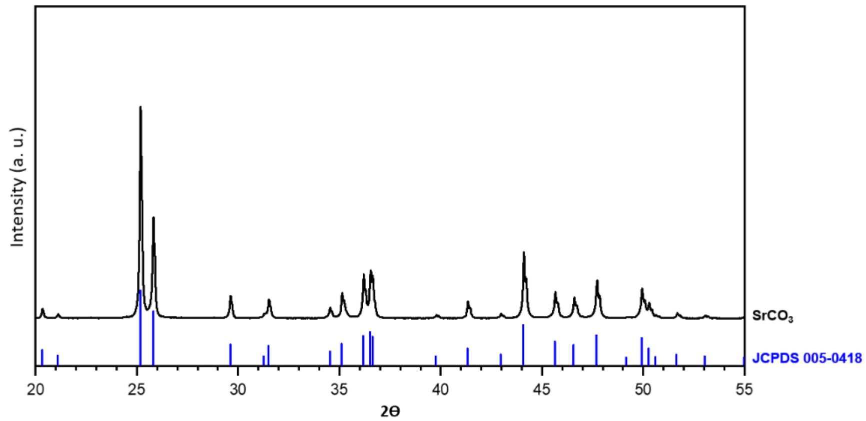

Figure S6. Typical XRD pattern of $SrCO_3$ (Alfa Aesar, USA).

TEM analysis of $SrCO_3$ nanorods

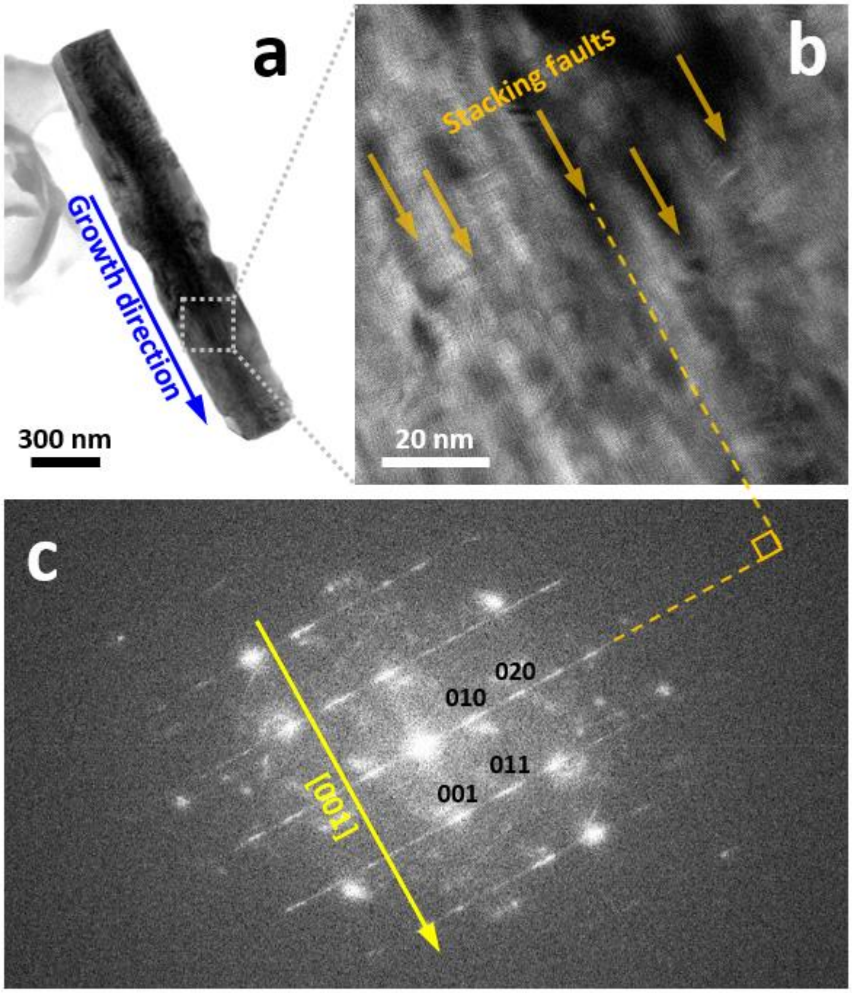

Figure S7. (a) TEM image of a $SrCO_3$ nanorod. (b) High-resolution TEM image of a selected area of the nanorod. (c) FFT diffraction pattern corresponding to Figure S7b, indexed to the orthorhombic $SrCO_3$ (*Pmcn*; JCPDS No. 05–0418). Supplementary Figure S7a shows a single $SrCO_3$ nanorod obtained by exposing $Sr(OH)_2{\cdot}8H_2O$ to humid condition. Supplementary Figure S7b shows a high-resolution (HR) TEM image where fringes, parallel to the growth direction of the nanorod (marked by arrows), exist, indicating planar defects. The corresponding FFT pattern is displayed in Supplementary Figure S7c. The diffraction spots are indexed to the orthorhombic $SrCO_3$ (*Pmcn*; JCPDS No. 05–0418) where the streaks, lying parallel to the [010] direction, reflect local disorder of the atomic stacking (i.e., stacking faults) as being perpendicular to the fringes in the HR-TEM image. The distance between the streaks, normal to the growth direction, corresponds to the (001) plane, i.e., 0.60 nm, demonstrating the growth direction of the nanorod to be <001>.

Attribution of Raman peaks

Table S2. Raman shift and related vibration modes in $SrCO_3$.

Raman shift (cm^{-1})	Vibration mode	Ref.
3000-3700	OH stretching of liquid water	20,21
3594, and 3609	OH stretching in $SrO_{0.9}(OH)_{0.3}$	22,23
3494	OH stretching in $Sr(OH)_2{\cdot}nH_2O$ $(1 \leq n \leq 8)$	23,24
3300, and 3384	OH stretching in $Sr(OH)_2{\cdot}8H_2O$	24
1073	Symmetric stretching of C–O in $SrCO_3$	25
~1055	Symmetrical stretching of C–O in CO_3^{2-} (aq)	26,27
866	O–O stretching in $Sr(O_2)_{0.98}O_{0.02}$	28,29
841, 903, and 953	O–O stretching in peroxide ions (O_2^{2-}) of $Sr(O_2)_{1-x}O_x$	30–32
701	in-plane bending of C–O in $SrCO_3$	33,34
539, and 518	Sr–O stretching in SrOH	35
396,* and 361	Sr–O–H bending in SrOH	35
244	external vibration between the cation and anionic groups	36
182, and 149	Angular oscillations of CO_3 ions	37

*The signal at 396 cm^{-1} is likely to stem from Sr–O–H signal at 361 cm^{-1}, probably because of the influence of surrounding CO_3^{2-} ions on the chemical environment of SrOH.

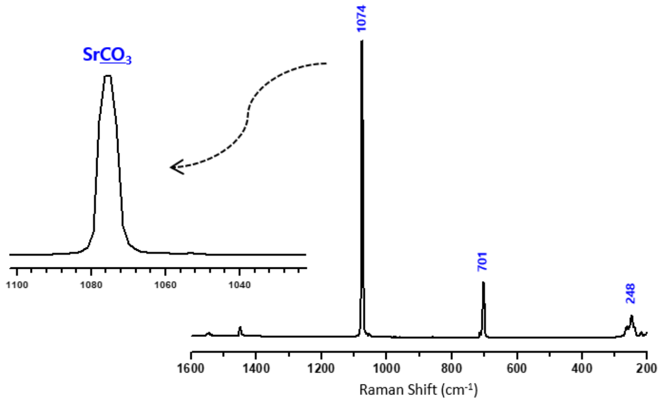

Figure S8. Typical Raman spectrum of $SrCO_3$ (Alfa Aesar, USA).

Relationship of pH and carbonate-ion concentrations

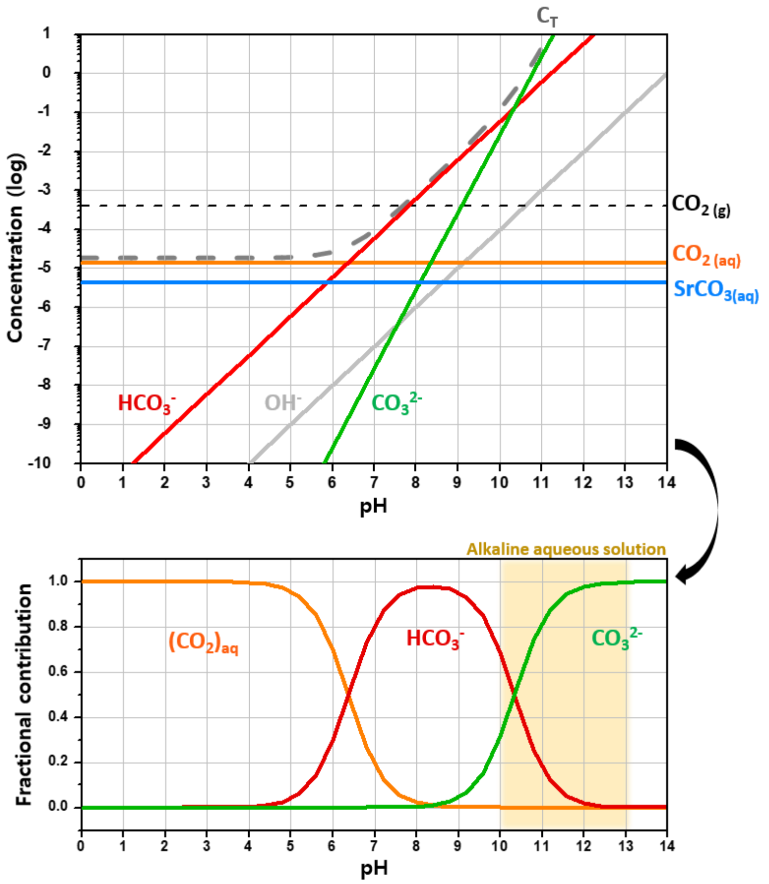

Figure S9. Bjerrum plot showing the concentration and fraction of [CO_2], [HCO_3^-], and [CO_3^{2-}] to the total dissolved inorganic carbon (C_T) in water as a function of pH, plotted using the database in HSC Chemistry 6.

In order to understand the $SrCO_3$ formation by reaction of strontium with carbon dioxide in alkaline aqueous solution, the concentration and dominant phase of inorganic carbon dissolvable in water are investigated. Supplementary Figure S9 (Top) shows concentrations of $CO_{2\,(aq)}$, $HCO_3^-{}_{(aq)}$, $CO_3^{2-}{}_{(aq)}$, $CO_{2\,(g)}$, and $SrCO_{3\,(aq)}$ as a function of pH. The relative proportions of [CO_2], [HCO_3^-], and [CO_3^{2-}] to the total dissolved inorganic carbon (C_T) in water with 0–14 pH are also drawn in Supplementary Figure S9 (bottom). It appears that the equilibrium concentrations of CO_3^{2-} and HCO_3^- ions exponentially increase as the concentration of OH^- (i.e., pH) increases, and vice versa (Supplementary Figure S9: Top). In other words, at elevated pH, the dissolved CO_2 is supposed to convert to HCO_3^- and CO_3^{2-} (Supplementary Figure S9: Bottom). Particularly above ~10.5 pH, the dominant carbon phase is found to be CO_3^{2-}. Since alkaline-earth-metal hydroxides have strong basicity (e.g., pH = 11.27–13.09 at 1–100 mM $Sr(OH)_2$), the CO_2 absorbed onto hydrated Sr would convert to CO_3^{2-} as per the reactions: $CO_2 + OH^- \leftrightarrow HCO_3^-$, and then $HCO_3^- + OH^- \leftrightarrow H_2O + CO_3^{2-}$. This interpretation is in good agreement with the results of Raman spectroscopy (Figure 5), where the increase of [CO_3^{2-}] is indeed observed, indicative of the CO_2 absorption.

Proposed reaction process for $SrCO_3$ formation

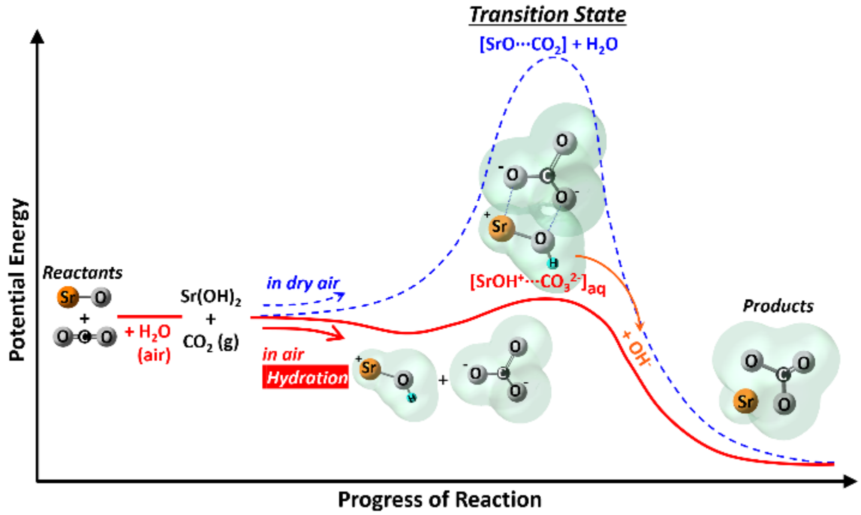

Figure S10. Proposed reaction process for the formation of $SrCO_3$ from SrO hydrated at room temperature: possible reaction sequence (solid red line), and commonly known reaction sequence (blue dashed line).

The influence of assembling time on the anisotropic growth of $SrCO_3$

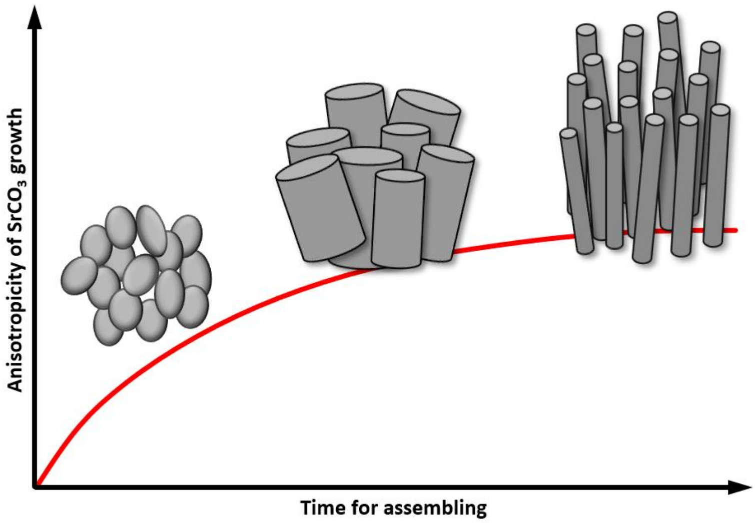

Figure S11. A simplified curve showing the effect of assembling time on the anisotropic growth of $SrCO_3$. The carbonation of SrO via hydration occurs following the reaction: $Sr(OH)_2 \cdot 8H_2O + CO_2 \rightarrow SrCO_3 + 9H_2O$. It is thus implied that the kinetics of $SrCO_3$ formation is affected by CO_2 and H_2O partial pressures (humidity level). Experimental results (Figures 2 and 3) have shown a trend for the morphology of $SrCO_3$ produced at the assembling time. The $SrCO_3$, produced under high humidity level and low CO_2 partial pressure (i.e., slow carbonation reaction), has nanorod morphology whereas the $SrCO_3$, grown under low humidity level and high CO_2 partial pressure (i.e., fast carbonation reaction), has spherical morphology. That is, as the reaction kinetics for the $SrCO_3$ formation becomes slower, the diameter of $SrCO_3$ nanorods becomes smaller in better alignment.

References

1. Sreedhar, B., Sulochana, M., Vani, C. S., Devi, D. K. & Naidu, N. V. S. Shape Evolution of Strontium Carbonate Architectures Using Natural Gums As Crystal Growth Modifiers. *Eur. Chem. Bull.* **3**, 234–239 (2014).

2. Alavi, M. A. & Morsali, A. Syntheses and characterization of $Sr(OH)_2$ and $SrCO_3$ nanostructures by ultrasonic method. *Ultrason. Sonochem.* **17**, 441–446 (2010).

3. Yu, J., Guo, H. & Cheng, B. Shape evolution of $SrCO_3$ particles in the presence of poly-(styrene-alt-maleic acid). *J. Solid State Chem.* **179**, 800–803 (2006).

4. Li, S., Zhang, H., Xu, J. & Yang, D. Hydrothermal synthesis of flower-like $SrCO_3$ nanostructures. *Mater. Lett.* **59**, 420–422 (2005).

5. Guo, G. S., Gu, F. B., Wang, Z. H. & Guo, H. Y. Low-temperature Growth of Single-crystal $SrCO_3$ Nanoneedles. *Chinese Chem. Lett.* **16**, 1101–1104 (2005).

6. Jahangiri, H., Ranjbar, M., Taher, M. A. & Kazerooni, H. Using Microwave Heating for Synthesis of $SrCO_3$ Nanostructures with Different Morphologies. *J. Ind. Eng. Chem.* **21**, 1132–1136 (2015).

7. Cao, M., Wu, X., He, X. & Hu, C. Microemulsion-Mediated Solvothermal synthesis of $SrCO_3$ Nanostructures. *Langmuir* **21**, 6093–6096 (2005).

8. Arumugam, D. *et al.* Growth Mechanism of Pine-leaf-like Nanostructure from the Backbone of $SrCO_3$ Nanorods using LaMer's Surface Diffusion: Impact of Higher Surface Energy (γ = 38.9 eV/nm^2) {111} Plane Stacking Along ⟨110⟩ (γ = 3.4 eV/nm^2) by First-Principles Calculations. *Cryst. Growth Des.* **17**, 6394–6406 (2017).

9. Yang, L., Chu, D., Wang, L., Ge, G. & Sun, H. Facile synthesis of porous flower-like $SrCO_3$ nanostructures by integrating bottom-up and top-down routes. *Mater. Lett.* **167**, 4–8 (2016).

10. Wang, W. S., Zhen, L., Xu, C. Y., Yang, L. & Shao, W. Z. Room temperature synthesis of hierarchical $SrCO_3$ architectures by a surfactant-free aqueous solution route. *Cryst. Growth Des.* **8**, 1734–1740 (2008).

11. Rautaray, D., Sanyal, A., Adyanthaya, S. D., Ahmad, A. & Sastry, M. Biological synthesis of strontium carbonate crystals using the fungus Fusarium oxysporum. *Langmuir* **20**, 6827–6833 (2004).

12. Guo, G., Yan, G., Wang, L. & Huang, J. Crystallization of strontium carbonate in alcohol or water solution containing mixed nonionic/anionic surfactants. *Mater. Lett.* **62**, 4018–4021 (2008).

13. Liao, F., Zhao, L., Zhai, C., Zhang, Z. & Ma, X. Morphology and photoluminescence properties of $SrCO_3$ prepared by a simple solution method. *Mater. Lett.* **122**, 331–333 (2014).

14. Wang, Z., He, G., Yin, H., Bai, W. & Ding, D. Evolution of controllable urchin-like $SrCO_3$ with enhanced electrochemical performance via an alternative processing. *Appl. Surf. Sci.* **411**, 197–204 (2017).

15. Zhang, W., Yu, Y. & Yi, Z. Controllable synthesis of $SrCO_3$ with different morphologies and their co-catalytic activities for photocatalytic oxidation of hydrocarbon gases over TiO_2. *J. Mater. Sci.*

52, 5106–5116 (2017).

16. Li, J. M. Realizing single-crystalline vertically-oriented and high-density electrospun nanofibril bundles by controlled postcalcination. *CrystEngComm* **19**, 3392–3397 (2017).

17. Aphale, A. *et al.* Synthesis and Stability of $Sr_xNi_yO_z$ Chromium Getter for Solid Oxide Fuel Cells. *J. Electrochem. Soc.* **165**, F635–F640 (2018).

18. Aphale, A., Hong, J., Hu, B. & Singh, P. Development and Validation of Chromium Getters for Solid Oxide Fuel Cell Power Systems. *J. Vis. Exp.* **147**, e59623 (2019).

19. Palmer, D. C. *CrystalDiffract User's Guide*. (CrystalMaker Software Ltd , UK, 2015).

20. Sun, Q. The Raman OH stretching bands of liquid water. *Vib. Spectrosc.* **51**, 213–217 (2009).

21. Carey, D. M. & Korenowski, G. M. Measurement of the Raman spectrum of liquid water. *J. Chem. Phys.* **108**, 2669–2675 (1998).

22. Slodczyk, A., Tran, C. & Colomban, P. Face to face with enemy – analysis of aqua carbonate hydroxide second surface phases in proton conducting perovskite ceramic electrolytic membrane. *Mater. Res. Soc. Symp. Proc.* **1384**, (2012).

23. Colomban, P., Tran, C., Zaafrani, O. & Slodczyk, A. Aqua oxyhydroxycarbonate second phases at the surface of Ba/Sr-based proton conducting perovskites: A source of confusion in the understanding of proton conduction. *J. Raman Spectrosc.* **44**, 312–320 (2013).

24. Lutz, H. D., Eckers, W., Schneider, G. & Haeuseler, H. Raman and infrared spectra of barium and strontium hydroxides and hydroxide hydrates. *Spectrochim. Acta Part A Mol. Spectrosc.* **37**, 561–567 (1981).

25. Wehrmeister, U., Soldati, A. L., Jacob, D. E., Häger, T. & Hofmeister, W. Raman spectroscopy of synthetic, geological and biological vaterite: A Raman spectroscopic study. *J. Raman Spectrosc.* **41**, 193–201 (2010).

26. Wen, N. & Brooker, M. H. Ammonium carbonate, ammonium bicarbonate, and ammonium carbamate equilibria: A raman study. *J. Phys. Chem.* **99**, 359–368 (1995).

27. Idris, Z., Jens, K. J. & Eimer, D. A. Speciation of MEA-CO_2 adducts at equilibrium using raman spectroscopy. *Energy Procedia* **63**, 1424–1431 (2014).

28. Upasen, S., Batocchi, P., Mauvy, F., Slodczyk, A. & Colomban, P. Chemical and structural stability of $La_{0.6}Sr_{0.4}Co_{0.2}Fe_{0.8}O_{3-\delta}$ ceramic vs. medium/high water vapor pressure. *Ceram. Int.* **41**, 14137–14147 (2015).

29. de Waal, D., Range, K.-J., Königstein, M. & Kiefer, W. Raman spectra of the barium oxide peroxide and strontium oxide peroxide series. *J. Raman Spectrosc.* **29**, 109–113 (1998).

30. Xie, S., Mestl, G., Rosynek, M. P. & Lunsford, J. H. Decomposition of Nitric Oxide over Barium Oxide Supported on Magnesium Oxide. 1. Catalytic Results and in Situ Raman Spectroscopic Evidence for a Barium-Nitro Intermediate. *J. Am. Chem. Soc.* **119**, 10186–10191 (1997).

31. Eysel, H. H. & Thym, S. RAMAN Spectra of Peroxides. *J. Inorg. Gen. Chem.* **411**, 97–102 (1975).

32. Mestl, G., Rosynek, M. P. & Lunsford, J. H. Decomposition of Nitric Oxide over Barium Oxide

Supported on Magnesium Oxide. 4. In Situ Raman Characterization of Oxide Phase Transitions and Peroxide Species by 18 O-Labeling. *J. Phys. Chem. B* **102**, 154–161 (1998).

33. Bonales, L. J. *et al.* Quantitative Raman spectroscopy as a tool to study the kinetics and formation mechanism of carbonates. *Spectrochim. Acta Part A Mol. Biomol. Spectrosc.* **116**, 26–30 (2013).

34. Tavender, S. M., Johnson, S. A., Balsom, D., Parker, A. W. & Bisby, R. H. The carbonate, Co3-., in solution studied by resonance Raman spectroscopy. *Laser Chem.* **19**, 311–316 (1999).

35. Brazier, C. R. & Bernath, P. F. Laser and fourier transform spectroscopy of the Ã2II-X̃2Σ+ transition of SrOH. *J. Mol. Spectrosc.* **114**, 163–173 (1985).

36. Buzgar, N. & Apopei, A. I. The Raman study of certain carbonates. *Geol. Tomul LV* **2**, 97–112 (2009).

37. Krishnan, T. S. Raman spectrum of strontianite ($SrCO_3$). *Proc. Indian Acad. Sci. - Sect. A* **44**, 96–98 (1956).
